# Challenges for future directions for artificial intelligence integrated nursing simulation education

**DOI:** 10.4069/kjwhn.2023.09.06.1

**Published:** 2023-09-26

**Authors:** Sunyoung Jung

**Affiliations:** College of Nursing and Research Institute of Nursing Science, Daegu Catholic University, Daegu, Korea

**Keywords:** Artificial intelligence, Nursing, Simulation training

## Abstract

Artificial intelligence (AI) has tremendous potential to change the way we train future health professionals. Although AI can provide improved realism, engagement, and personalization in nursing simulations, it is also important to address any issues associated with the technology, teaching methods, and ethical considerations of AI. In nursing simulation education, AI does not replace the valuable role of nurse educators but can enhance the educational effectiveness of simulation by promoting interdisciplinary collaboration, faculty development, and learner self-direction. We should continue to explore, innovate, and adapt our teaching methods to provide nursing students with the best possible education.

## Introduction

Artificial intelligence (AI) refers to the development of computer systems capable of performing tasks that have typically required human intelligence, such as visual perception, natural language processing, and decision-making [1]. Various applications of AI are found in healthcare, including analysis of medical imaging, clinical decision-making support systems, virtual nursing assistants, and patient monitoring [2]. Nursing simulation education uses scenarios, equipment, and techniques to replicate real-world healthcare situations and provide a safe environment for students to develop clinical skills, critical thinking, and decision-making abilities [3].

AI is currently integrated into nursing simulation education to enhance realism and interactivity and to personalize the learning experience for students. It has the potential to revolutionize the way we educate future nurses [4]. AI in nursing simulation education is the integration of AI technologies and methodologies into nursing simulations to enhance the learning experience and improve outcomes for nursing students. AI can be applied to various aspects of nursing simulation including virtual patient models, intelligent debriefing systems, adaptive learning platforms, and clinical decision support systems [4-7].

First, AI technologies can be used to develop virtual patient models that mimic real-life clinical scenarios. These virtual patients can exhibit realistic physiological responses, such as vital sign changes, symptoms, and behaviors, based on input from the student’s actions. AI algorithms enable virtual patients to adapt their responses dynamically, providing a more realistic and interactive simulation experience [8]. Another advantage is that learners can conduct simulations without having to come to the training site and can initiate self-study without the instructor directly operating the simulation. Second, AI can be utilized to create intelligent debriefing systems that provide personalized feedback and guidance to nursing students. These systems can analyze the student’s actions and decisions during the simulation and offer tailored feedback, highlighting areas of strength and areas for improvement. AI algorithms can also identify patterns in student performance and provide targeted remediation strategies [9]. Third, AI-driven adaptive learning platforms can customize the learning experience to each student’s individual needs, knowledge level, and learning style. Competition in technological development is accelerating as information technology companies such as Apple, Google, and Microsoft supply their big data-based and AI learning solutions to schools and online learning services. These platforms can analyze data on student performance, identify knowledge gaps, and deliver targeted educational content or simulation scenarios to address those gaps. Adaptive learning platforms can help optimize the learning process, ensuring that students receive the most relevant and effective educational materials [4,10,11]. Fourth, AI algorithms can be integrated into nursing simulation scenarios to provide clinical decision-making support to students. These systems can analyze patient data, simulate various treatment options, and offer recommendations based on evidence-based guidelines. Clinical decision-making support systems can help students develop critical thinking and clinical judgment skills by exposing them to complex and realistic patient-care scenarios [4,11].

The aim of this review was to understand the potential benefits, limitations, ethical considerations, and challenges of nursing simulation education with AI. In addition, the author would like to suggest an optimal direction for nursing simulation education with AI.

## The benefits of nursing simulation education with artificial intelligence

The application of AI in nursing simulation education offers several benefits [4]. However, each benefit must first be evaluated for compliance with the Healthcare Simulation Standards of Best Practice (HSSOPE^TM^) of the International Nursing Association for Clinical Simulation and Learning (INACSL) [11]. The first benefit is enhanced realism and fidelity. AI-driven simulations can provide realistic patient scenarios that replicate various physiological and psychological responses and create an immersive learning environment. The second benefit is improved student engagement and active learning. AI can engage students through interactive virtual patients, adaptive feedback, and gamification, all of which promote active participation and knowledge retention. This allows for the efficient use of time by students who are waiting for their turn with a simulation run directly by the professor (a problem in existing simulation education). Students can engage in preliminary learning while waiting their turn. The third benefit is personalized learning. AI algorithms can tailor simulations to each student’s individual learning needs by adjusting difficulty levels, providing personalized feedback, and tracking progress. The final benefit is an efficient student evaluation and feedback mechanism. AI-based assessment tools automatically analyze student performance, provide objective feedback, and enable self-debriefing through self-reflection. In addition, AI simulations automatically generate evaluation reports on each student’s nursing performance, saving instructors time in writing reports and improving evaluation accuracy [4,8-11].

These benefits met the criteria of HSSOPE^TM^ from the INACSL. Simulation with AI was found to be adaptable because these benefits improved the modality, fidelity, and enhanced facilitation of nursing simulation design, outcomes, and objectives for nursing simulation education [12,13]. In addition, it enhanced the debriefing time with individualized feedback, and that data can be used to develop various methods of learner evaluation after simulation. Thus, it can be said to meet the criteria for debriefing, facilitation, and evaluation [11,14].

## Challenges and hurdles

There are technical challenges for nursing simulation education with AI. The first technical challenge is the integration of AI systems into existing simulation infrastructure. Adapting AI technologies to work seamlessly with current simulation equipment and software can be complex and require substantial resources [4,7,10]. The second technical challenge is data privacy and security concerns. AI simulations use sensitive patient data, raising ethical and legal considerations regarding data privacy, security, and confidentiality [5-9]. The third challenge is cost and resource allocation. Implementing AI-driven simulation education may require a significant financial investment, including acquisition of AI tools, training faculty, and maintaining technical infrastructure [4-7,9,10].

In addition to technical challenges, there are pedagogical challenges in nursing simulation education with AI. The first pedagogical challenge is designing effective AI-driven simulations. Developing high-quality AI simulations necessitates collaboration between nursing educators and AI experts to ensure clinical accuracy, realism, and alignment with learning objectives [5,8,10]. The second challenge is ensuring cultural sensitivity and inclusivity. AI algorithms and simulations should be sensitive to diverse patient populations, respecting cultural, ethnic, and socioeconomic differences to provide equitable learning experiences [4,6,7,10]. The third challenge is balancing AI reliance with critical thinking and the development of clinical judgment. While AI can provide valuable support, it is crucial to maintain a balance between AI assistance and fostering critical thinking skills, clinical reasoning, and ethical decision-making in nursing students [4-6,8-10].

## Ethical considerations in artificial intelligence-driven nursing simulation education

While the advantages and development potential of nursing simulation education with AI are infinite, risk factors such as AI hallucination are also possible. Therefore, we must set ethical principles and guidelines [4]. Ethical considerations include autonomy and patient privacy. AI simulations must prioritize patient autonomy and privacy by ensuring informed consent, protecting sensitive data, and addressing potential risks associated with the use of AI [4,8-10]. A second ethical consideration is the potential impact on the role of nursing educators. AI integration may necessitate redefining the role of nursing educators, emphasizing their expertise in guiding students’ learning, ethical decision-making, and professional development [4,8-10]. A third ethical challenge is addressing bias in AI algorithms and simulations. It is crucial to identify and mitigate biases within AI algorithms (e.g., a facial recognition algorithm may be trained to recognize white people more easily than black people because this type of data was used more frequently for training in machine learning) to ensure fair and unbiased representation of diverse patient populations in simulations [4,8-10]. Finally, transparency and accountability in AI systems is an ethical challenge. AI-driven simulations should provide transparency regarding data sources, algorithms, and decision-making processes, enabling users to understand and question the system’s outputs [4-10]. In addition, all educators and students must adhere to ethical standards and confidentiality, as emphasized by the professional integrity criteria of HSSOPE^TM^ from the INACSL [15].

## Strategies to overcome challenges

Strategies to overcome the challenges in nursing simulation with AI include (1) collaboration between nursing educators and AI developers, (2) faculty development programs for AI integration, (3) rigorous evaluation and research on AI-driven simulations, and (4) engaging students in the dialogue on AI in nursing education [4 6,8-10].

To live in the age of AI, we must remain competitive with AI as suggested by Lee [16] in the acronym PROMPT: Planning (and prospect), Reconstruction, Organize, Make a question, Persuasion, Together (and touching). By using this PROMPT method, we can apply AI-powered language models (e.g., ChatGPT) and AI to our daily lives. In addition, the PROMPT method can contribute to more effective and creative nursing education. For example, the field of women’s health nursing emphasizes the need for individualized nursing care through communication with patients preparing for childbirth. By using the PROMPT method as a colleague as well as a tool in the field of women’s health nursing and women’s health nursing simulation education, we can contribute to more effective and creative nursing education.

## Conclusion

The future is already here, and AI can pave the way by augmenting, not replacing, the valuable role of nursing educators. Given the challenges and ethical issues in integrating AI into education, we must continue to explore, innovate, and adapt our teaching methods to provide nursing students with the best possible education.
